# Development and Validation of a Decent Work Scale for Hospital Nurses: An Experiential Perspective on Working Conditions

**DOI:** 10.3390/nursrep16070237

**Published:** 2026-07-09

**Authors:** Yasue Yamazumi, Sachiko Tanaka

**Affiliations:** 1 Faculty of Nursing, Kyoritsu Women’s University, Tokyo 101-0051, Japan; 2 School of Nursing, The Jikei University, Tokyo 105-8461, Japan; satanaka@jikei.ac.jp

**Keywords:** decent work, nurses, scale development, psychometric evaluation, work engagement, burnout, job crafting, labour rights, nursing workforce

## Abstract

**Background/Objectives**: Nursing is characterised by demanding working conditions, including long working hours and staffing shortages. Although Decent Work (DW) has been proposed as a framework for improving labour conditions, existing measures do not adequately capture how these conditions are experienced in practice. This study aimed to develop and validate the Decent Work Scale for Hospital Nurses (DWS-N), conceptualising DW as the experiential realisation of structural labour conditions. **Methods**: A multi-phase methodological design was employed. In Phase 1, 184 items were generated through concept analysis and refined to 142 items via expert review. In Phase 2, exploratory and confirmatory factor analyses were conducted to examine structural validity and reliability. In Phase 3, cross-validation and criterion-related validity were assessed using an independent sample. **Results**: A five-factor structure comprising 22 items was identified: Meaningful and Fulfilling Work, Protection of Workers’ Rights, Supportive and Safe Working Environment, Appropriate Working Time and Work–Life Balance, and Adequate Compensation. The scale demonstrated good internal consistency (Cronbach’s α = 0.896) and acceptable fit (CFI = 0.969, RMSEA = 0.037). Cross-validation supported model stability (CFI = 0.917, RMSEA = 0.070). DW was positively associated with work engagement and negatively associated with burnout. Different dimensions of DW showed distinct patterns of association with occupational outcomes. **Conclusions**: The DWS-N is a valid and reliable instrument for assessing Decent Work in nursing. By capturing the experiential realisation of structural labour conditions, the scale offers a context-specific approach to understanding nurses’ working environments, with potential applications in workforce management and organisational policy.

## 1. Introduction

Nursing represents one of the largest professional groups in healthcare systems worldwide and is characterised by shared structural labour conditions, including staffing shortages, long working hours, shift work, emotional labour and exposure to occupational risks [1,2,3]. These challenges are further shaped by gendered labour structures, as nursing remains a predominantly female profession globally and has historically been associated with undervaluation, role stereotyping and limited recognition of professional autonomy. These conditions are associated with adverse outcomes such as burnout, job dissatisfaction, turnover intention and reduced quality of care [1,4,5,6]. Large-scale international studies have further demonstrated that poor work environments are linked to patient safety outcomes, including increased mortality and adverse events [1,2]. More recent evidence has continued to demonstrate that supportive nursing work environments are associated with better patient outcomes, improved quality of care, and lower levels of nurse burnout [7,8,9]. These findings indicate that nurses’ working conditions are not merely an occupational issue but a critical determinant of healthcare system performance.

From a theoretical perspective, nursing work has frequently been examined using frameworks such as the Job Demands–Resources (JD-R) model, which conceptualises excessive job demands as sources of strain and insufficient job resources as barriers to engagement and well-being [10,11,12]. Classical motivation theories have similarly emphasised the importance of working conditions in shaping motivation and job satisfaction [13]. However, much of the existing nursing literature has focused on individual-level psychological outcomes, including burnout and work engagement [14,15,16]. Following the COVID-19 pandemic, concerns regarding healthcare workforce well-being have intensified, and maintaining healthy work environments has become a global priority for healthcare systems [17,18]. Although these constructs provide important insights into nurses’ responses to work, they do not fully address the structural and normative dimensions of work, such as labour rights, fairness, employment conditions and social protection.

The concept of Decent Work (DW), proposed by the International Labour Organization (ILO), offers a comprehensive framework for understanding the quality of work and its implications for well-being and sustainable development [19,20,21,22,23,24]. DW encompasses multiple dimensions, including employment opportunities, adequate earnings, working time, occupational safety, social protection and workers’ rights [19,20,21]. Recent theoretical developments have emphasised that DW should be understood not only in terms of formal institutional arrangements but also in terms of how these conditions are realised in workers’ lived experiences [25,26,27]. This perspective is particularly relevant in nursing, where organisational constraints, staffing systems and professional norms may create a gap between formal policies and actual work practices. Because nurses’ ability to provide safe and high-quality care depends not only on formal employment conditions but also on how these conditions are experienced in daily practice, Decent Work may represent an important foundation for sustaining both workforce well-being and quality of patient care.

Despite growing interest in DW, its empirical assessment has largely relied on instruments developed for general working populations. The Decent Work Scale (DWS) and the Decent Work Questionnaire (DWQ) have demonstrated acceptable psychometric properties [28,29], but they do not fully capture the organisational characteristics and institutional constraints that shape nursing practice. In addition, these instruments primarily assess the presence of favourable conditions rather than how such conditions are experienced and enacted in everyday work. Previous studies in nursing have highlighted the discrepancy between formal working arrangements and the conditions that enable nurses to continue working [30]. Given the highly structured nature of nursing work, characterised by shift work, hierarchical team structures and regulated staffing systems, there is a need for a profession-specific approach to assessing DW. Furthermore, contemporary perspectives on job crafting suggest that employees actively shape their work experiences within structural constraints [31,32]. Recent studies have shown that higher levels of job crafting among nurses are associated with greater work engagement [33]. Understanding how structural working conditions influence such proactive work-related behaviours may provide further insight into nurses’ adaptation to demanding work environments.

Taken together, these considerations suggest the need for a context-specific and psychometrically robust instrument that conceptualises Decent Work as the experiential realisation of structural working conditions in nursing practice. Given the persistent workforce crisis and increasing emphasis on healthcare workforce well-being, a profession-specific instrument is needed to better understand and improve nurses’ working conditions. Therefore, the aim of this study was to develop and validate a Decent Work Scale for hospital nurses (DWS-N) using a multi-phase methodological design to capture the experiential realisation of structural working conditions in nursing practice.

## 2. Materials and Methods

### 2.1. Study Design and Procedures

This study employed a multi-phase methodological design to develop and validate the Decent Work Scale for Nurses (DWS-N). The study was grounded in the conceptual framework of Decent Work (DW) proposed by the International Labour Organisation (ILO), which defines DW as work that ensures fair income, security in the workplace, social protection, and rights at work [19,20,21]. The study design followed the COSMIN (COnsensus-based Standards for the selection of health Measurement INstruments) guidelines [34] and established best practices for scale development and psychometric evaluation [35,36], ensuring methodological rigor and transparency.

In this study, DW for nurses was operationally defined as a state in which workers’ rights are protected, individuals receive equal treatment in an appropriate working environment, and opportunities for self-actualiation are achieved through fulfilling work. This definition reflects both structural labour conditions and their experiential realisation in nursing practice.

The research was conducted sequentially across three phases. In Phase 1, item generation and content validation were performed. In Phase 2, structural validity and reliability were examined. In Phase 3, cross-validation and criterion-related validity were evaluated using an independent sample.

During Phase 1, an initial pool of 184 items was generated based on a concept analysis of DW in nursing using the method proposed by Walker and Avant [37]. The concept analysis was informed by 42 literature sources (13 Japanese and 29 international publications), including ILO frameworks [19,20,21] and previous measurement studies [28,29]. Items were constructed to reflect both macro-level structural conditions (e.g., institutional policies and labour protections) and micro-level experiential aspects (e.g., perceived fairness, work meaning, and daily practice conditions). Content validity was evaluated through a structured expert panel review consisting of clinical nurses (Clinical Ladder levels II–V), certified nursing managers, and academic researchers with expertise in nursing science and labour-related research. Consistent with recommendations for preserving conceptual richness in complex constructs [35], a qualitative consensus-building approach was adopted rather than relying solely on quantitative indices such as the Content Validity Index. Through iterative discussions, discrepancies were resolved and consensus was achieved, resulting in refinement of the item pool from 184 to 142 items.

### 2.2. Participants and Settings

Participants were hospital nurses working in large-scale acute care hospitals with 400 or more beds in Japan. These settings were selected because they provide relatively standardized organizational structures, including staffing systems, shift work arrangements, and regulatory frameworks. This enabled a consistent examination of the experiential realisation of DW within comparable institutional environments.

For Phase 2, data were collected between May and June 2025 using self-administered questionnaires. A total of 356 valid responses were obtained and included in the analysis. Although traditional recommendations for exploratory factor analysis often suggest recruiting five to ten participants per item, contemporary psychometric literature indicates that an absolute sample size exceeding 300 participants is generally considered robust and adequate for stable factor extraction [35,36,38]. To assess temporal stability, a subsample of participants (*n* = 127) completed the questionnaire twice over a four-week interval.

For Phase 3, an independent nationwide sample of 247 hospital nurses was recruited in September 2025 following additional ethical approval obtained on 1 August 2025, and data were collected from September to October 2025. The use of this independent sample enabled examination of the stability and generalizability of the established factor structure.

### 2.3. Statistical Analysis

All statistical analyses were conducted using IBM SPSS Statistics (Version 30; IBM Corp., Armonk, NY, USA) and IBM SPSS Amos (Version 28; IBM Corp., Armonk, NY, USA). To assess potential common method bias, Harman’s single-factor test was performed using unrotated principal component analysis [39].

In Phase 2, item analysis based on Classical Test Theory was conducted using skewness and kurtosis (±2.0), ceiling and floor effects (>15.0%), and item-total correlations (>0.30) [35]. Exploratory factor analysis (EFA) was performed using maximum likelihood estimation with promax rotation, assuming correlations among latent constructs of DW [40]. The number of factors was determined based on eigenvalues (>1.0), scree plot examination, and interpretability. Confirmatory factor analysis (CFA) evaluated model fit using the following criteria: Goodness-of-Fit Index (GFI ≥ 0.90), Adjusted Goodness-of-Fit Index (AGFI ≥ 0.85), Comparative Fit Index (CFI ≥ 0.90), and Root Mean Square Error of Approximation (RMSEA ≤ 0.05) [41]. Internal consistency was assessed using Cronbach’s α coefficients (α ≥ 0.70), and temporal stability was evaluated using intraclass correlation coefficients (ICC ≥ 0.70) [42].

In Phase 3, cross-validation was conducted using CFA to verify the stability and generalizability of the factor structure in an independent sample [43]. Criterion-related validity was examined through associations with theoretically relevant constructs. Work engagement was measured using the Utrecht Work Engagement Scale (UWES) [15,16], burnout was assessed using the Copenhagen Burnout Inventory (CBI) [44,45], and job crafting was measured using the Japanese version of the Job Crafting Scale (JC) [32]. Based on previous studies [12,24,33], DW was hypothesized to be positively associated with work engagement and job crafting and negatively associated with burnout. Pearson correlation and multiple regression analyses were performed to examine these relationships.

### 2.4. Ethical Considerations and Use of Generative Artificial Intelligence

The study was conducted in accordance with the Declaration of Helsinki. Ethical approval was obtained from the Ethics Committee of The Jikei University School of Medicine for Phases 1 and 2 (Approval No. 36-448 (12568), date of approval 11 March 2025) and separately for Phase 3 (Approval No. 37-186 (12828), date of approval 1 August 2025), and from the Research Ethics Review Committee of Kyoritsu Women’s University and Kyoritsu Women’s Junior College (Approval No. 24019, date of approval 17 February 2025). Participation was voluntary, and consent was implied by the return of anonymous questionnaires.

During the preparation of this manuscript, the authors used a generative artificial intelligence tool (ChatGPT, OpenAI, GPT-4 series) for English-language editing and refinement. All outputs were carefully reviewed and edited by the authors, who take full responsibility for the accuracy, integrity, and content of the final manuscript.

## 3. Results

### 3.1. Content Validity (Phase 1)

Content validity was evaluated through responses and qualitative feedback from nine hospital nurses (Clinical Ladder levels II–V) and an expert panel consisting of researchers (*n* = 5), certified nurse managers (*n* = 2), senior nurses (*n* = 3) and a nurse with a master’s degree (*n* = 1). The panel assessed item comprehensibility, cultural relevance and clarity of expression.

As a result, 20 items were removed, 31 items were revised and 22 items were integrated. Through the integration of redundant items and subsequent reduction, the initial pool of 184 items was reduced to 142 items.

### 3.2. Structural Validity and Reliability (Phase 2)

#### 3.2.1. Item Analysis

A total of 1176 questionnaires were distributed, and 361 were returned (response rate: 30.7%). After excluding incomplete responses, 356 valid responses were included (valid response rate: 30.3%).

Mean item scores ranged from 2.12 to 4.80, and standard deviations ranged from 0.51 to 1.68. Eight items with skewness or kurtosis exceeding ± 2.0 and 22 items showing ceiling or floor effects (>15.0%) were removed. Additionally, 19 items were excluded based on item-total correlations (<0.30) and inter-item correlations (>0.75). Consequently, 93 items were retained for factor analysis.

#### 3.2.2. Sampling Adequacy

The Kaiser–Meyer–Olkin measure was 0.924, and Bartlett’s test of sphericity was significant (*χ*^2^ = 9061.92, df = 1128, *p* < 0.001), indicating suitability for factor analysis.

#### 3.2.3. Exploratory Factor Analysis

Exploratory factor analysis using maximum likelihood estimation with promax rotation identified a five-factor structure comprising 22 items. The cumulative variance explained was 50.99%, and inter-factor correlations ranged from 0.330 to 0.667 (*p* < 0.01). The five factors were labelled as Meaningful and Fulfilling Work, Protection of Workers’ Rights, Supportive and Safe Working Environment, Appropriate Working Time and Work–Life Balance, and Adequate Compensation. The strongest loading in each factor was observed for Item 113 (“My job enables me to utilize my strengths”) in Factor 1 (0.860), Item 93 (“My workplace respects individual opinions and positions”) in Factor 2 (0.872), Item 47 (“My hospital addresses mental health issues”) in Factor 3 (0.624), Item 45 (“I have sufficient time for hobbies and personal development”) in Factor 4 (0.806), and Item 1 (“I receive adequate pay for my work”) in Factor 5 (0.874). The factor structure of the final 22-item scale is presented in Table 1.

#### 3.2.4. Confirmatory Factor Analysis

Confirmatory factor analysis refined the model to 22 items across five factors.

The model fit indices were: *χ*^2^ = 297.36, df = 201, *χ*^2^/df = 1.48, GFI = 0.932, AGFI = 0.914, CFI = 0.969, RMSEA = 0.037 and RMR = 0.045.

**Figure 1:**
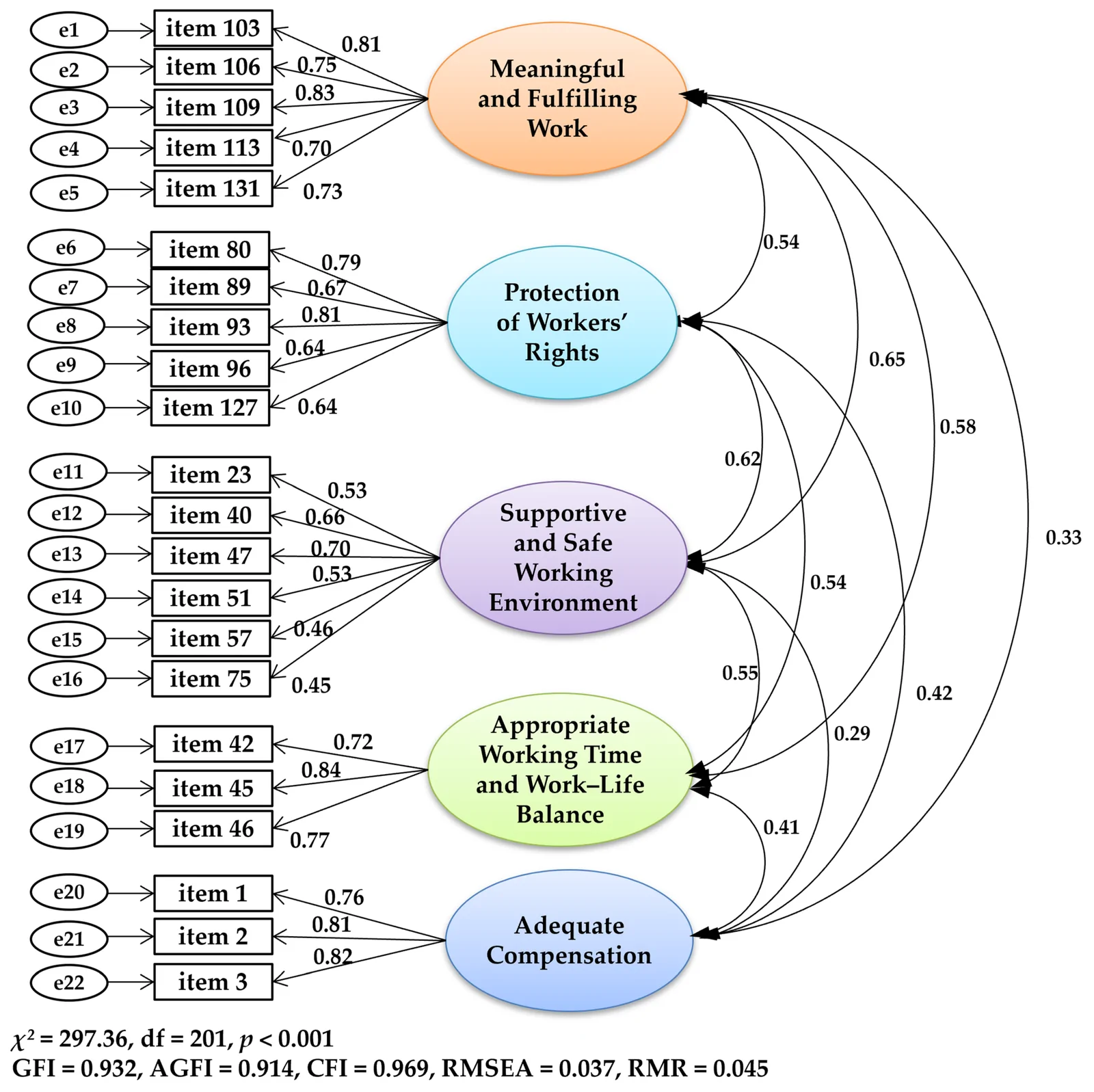
Confirmatory factor analysis model of the Decent Work Scale for Hospital Nurses (Phase 2). Abbreviations: GFI, Goodness-of-Fit Index; AGFI, Adjusted Goodness-of-Fit Index; CFI, Comparative Fit Index; RMSEA, Root Mean Square Error of Approximation; RMR, Root Mean Square Residual. Single-headed arrows indicate standardized factor loadings, double-headed curved arrows indicate correlations between latent factors, and e1–e22 indicate error terms.

The model is presented in Figure 1.

#### 3.2.5. Reliability

The mean total score was 72.6 ± 12.9. Cronbach’s α coefficients for the subscales ranged from 0.725 to 0.875. Test–retest reliability (*n* = 127) showed intraclass correlation coefficients ranging from 0.716 to 0.782.

### 3.3. Cross-Validation and Associations with Occupational Outcomes (Phase 3)

#### 3.3.1. Participant Characteristics

A total of 663 questionnaires were distributed, and 258 were returned (response rate: 38.9%). After excluding incomplete responses, 247 valid responses were included in the analysis (valid response rate: 37.3%). Participants were predominantly female (84.6%), with a mean age of 39.5 ± 11.1 years. Detailed characteristics are shown in Table 2.

#### 3.3.2. Cross-Validation: Confirmatory Factor Analysis

The total score was 74.0 ± 12.4.

Model fit indices were: *χ*^2^ = 435.66, df = 199, *χ*^2^/df = 2.19, GFI = 0.867, AGFI = 0.831, CFI = 0.917, RMSEA = 0.070 and RMR = 0.070.

**Figure 2:**
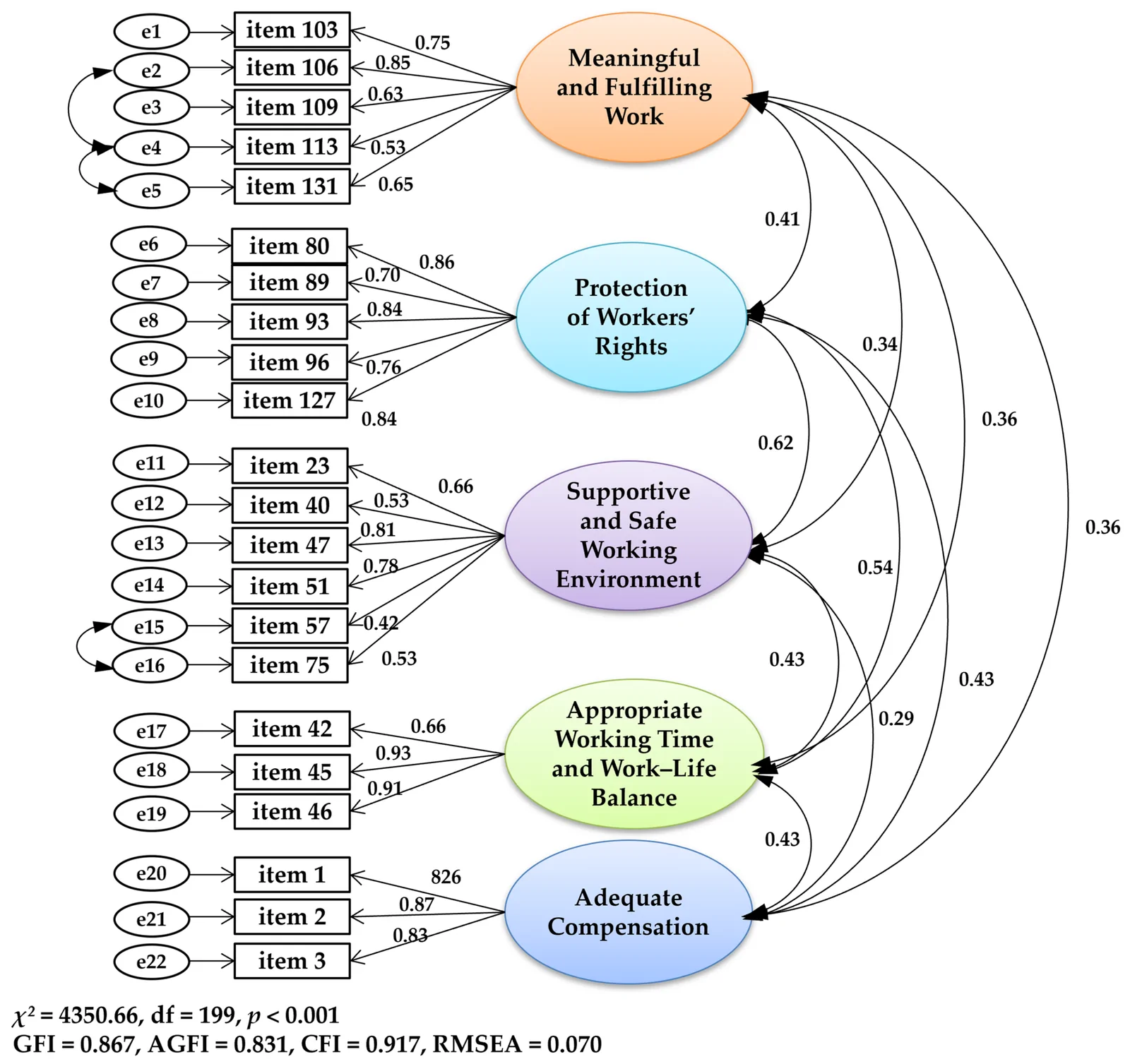
Cross-validation of the confirmatory factor analysis model of the Decent Work Scale for Hospital Nurses using an independent sample (Phase 3). Abbreviations: GFI, Goodness-of-Fit Index; AGFI, Adjusted Goodness-of-Fit Index; CFI, Comparative Fit Index; RMSEA, Root Mean Square Error of Approximation; RMR, Root Mean Square Residual. Single-headed arrows indicate standardized factor loadings, double-headed curved arrows between latent factors indicate correlations between latent factors, the double-headed curved arrow between e15 and e16 indicates error covariance, and e1–e22 indicate error terms.

The model is shown in Figure 2.

#### 3.3.3. Concurrent Validity

Decent Work scores showed a moderate positive correlation with work engagement (*r* = 0.458, *p* < 0.001), a moderate negative correlation with burnout (*r* = −0.533, *p* < 0.001), and a weak positive correlation with job crafting (*r* = 0.277, *p* < 0.001). Inter-factor correlations ranged from 0.320 to 0.400. Detailed results are presented in Table 3.

#### 3.3.4. Multiple Regression Analysis

**Figure 3:**
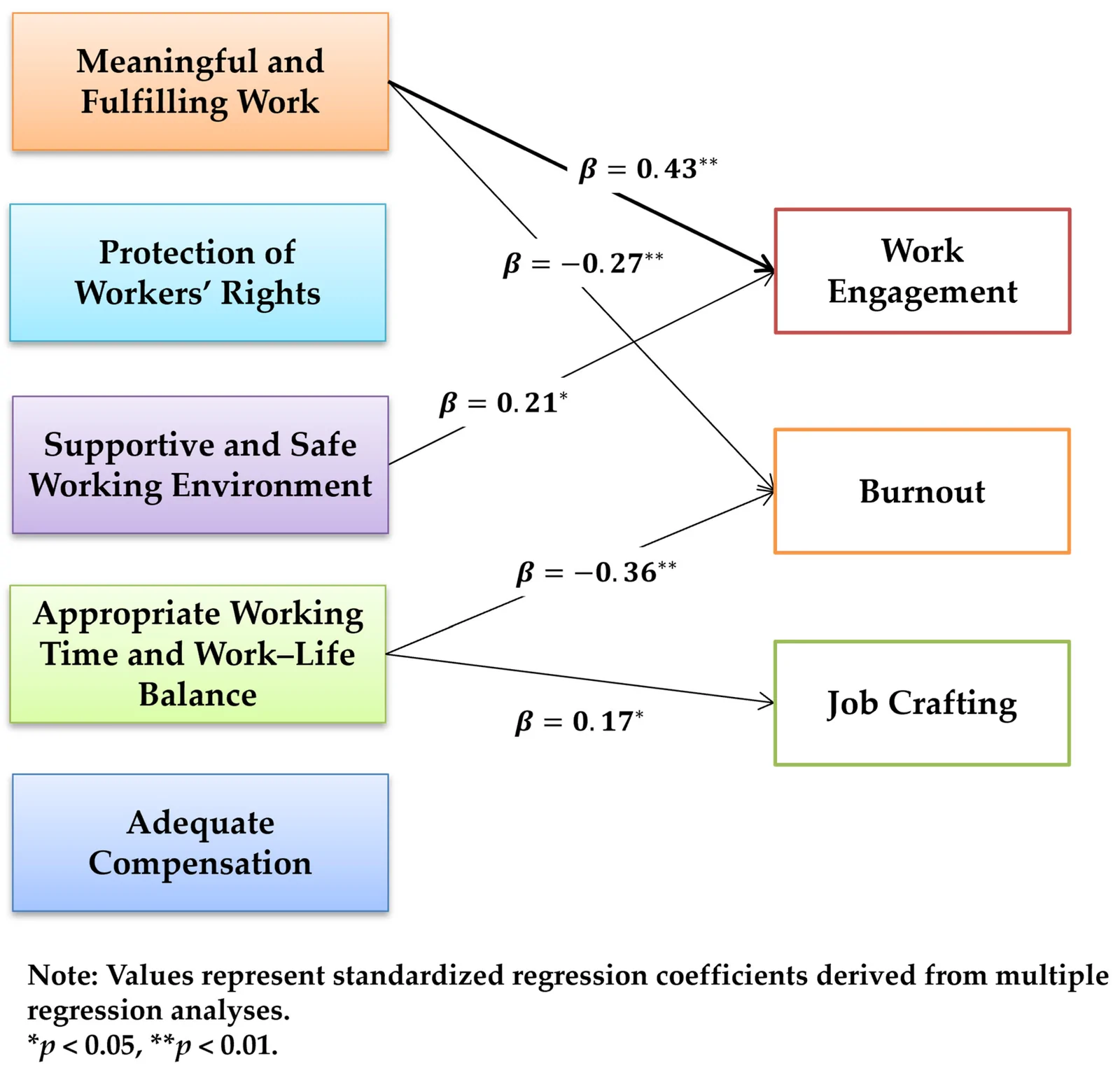
Structural model of the relationships between the DWS-N factors and occupational outcomes. Single-headed arrows indicate standardized regression coefficients, and thicker arrows indicate larger standardized regression coefficients (β).

Multiple regression analyses revealed that Meaningful and Fulfilling Work (*β* = 0.43, *p* < 0.001) and Supportive and Safe Working Environment (*β* = 0.21, *p* = 0.002) were significantly associated with work engagement, explaining 30% of the variance (R^2^ = 0.30). Regarding burnout, Meaningful and Fulfilling Work (*β* = −0.27, *p* < 0.001) and Appropriate Working Time and Work–Life Balance (*β* = −0.36, *p* < 0.001) were significantly associated, accounting for 36% of the variance (R^2^ = 0.36). For job crafting, Meaningful and Fulfilling Work (*β* = 0.20, *p* = 0.004) and Appropriate Working Time and Work–Life Balance (*β* = 0.17, *p* = 0.030) showed significant associations, explaining 11% of the variance (R^2^ = 0.11). Detailed regression coefficients are presented in Table 4, and the relationships between DWS-N factors and occupational outcomes are illustrated in Figure 3.

#### 3.3.5. Reliability (Phase 3)

Cronbach’s α for the total scale was 0.908. Subscale α values ranged from 0.787 to 0.899.

## 4. Discussion

### 4.1. Summary and Interpretation of Findings

This study developed and validated the Decent Work Scale for Hospital Nurses (DWS-N) using a multi-phase methodological design. The results demonstrated a stable five-factor structure, satisfactory internal consistency and test–retest reliability, and acceptable model fit across both the initial and cross-validation samples. In addition, the scale showed meaningful associations with theoretically relevant constructs, including work engagement, burnout, and job crafting, supporting its criterion-related validity [7,8,9,10,11,12,15,16].

Regarding structural validity, confirmatory factor analysis conducted in Phase 3 yielded slightly lower Goodness-of-Fit Index (GFI = 0.867) and Adjusted Goodness-of-Fit Index (AGFI = 0.831) values than conventional criteria. However, the Comparative Fit Index (CFI = 0.917) exceeded the recommended threshold and the Root Mean Square Error of Approximation (RMSEA = 0.070) indicated an acceptable level of fit according to widely accepted criteria [41]. Previous methodological studies have noted that GFI and AGFI are particularly sensitive to sample size and model complexity and may underestimate model fit in multidimensional models [38,41,43].

Similar challenges have been reported in previous studies validating multidimensional measures of Decent Work, including the Decent Work Scale and the Decent Work Questionnaire [28,29]. Because these instruments assess complex and theoretically rich constructs, achieving uniformly acceptable fit indices is inherently difficult. Nevertheless, these scales have demonstrated satisfactory psychometric properties and have subsequently been widely used in empirical research. Therefore, slight deviations in GFI and AGFI values should be interpreted in light of the overall pattern of fit indices and the theoretical coherence of the model rather than relying on a single criterion.

Taken together, these findings support the stability and generalizability of the five-factor structure of the DWS-N and suggest that the scale provides a psychometrically robust instrument for assessing Decent Work as experienced by hospital nurses.

### 4.2. Conceptual Contribution: Decent Work as Experiential Realisation

A key contribution of this study lies in reconceptualising Decent Work as the experiential realisation of structural labour conditions. While the International Labour Organization framework defines Decent Work in terms of institutional arrangements such as fair income, social protection, labour rights, and sustainable development [19,20,21,22], and previous studies have proposed statistical approaches to measuring Decent Work [23], the present findings suggest that these conditions should also be understood in terms of how they are perceived and enacted in everyday clinical practice.

Importantly, the DWS-N does not assess the mere presence of organisational provisions, such as welfare policies or formal labour protections, but rather the extent to which these provisions are actually experienced and realised by individual nurses. This distinction is important because favourable working conditions do not necessarily translate into improved well-being unless they are effectively realised in practice [24].

This perspective is consistent with recent developments in the psychology of working framework, which emphasise the dynamic interaction between structural conditions and workers’ lived experiences [25,26]. Furthermore, a psychosocial approach to Decent Work has highlighted the importance of understanding work not only as an objective institutional arrangement but also as a subjective and relational experience [27].

In nursing contexts, where staffing shortages, hierarchical structures, and organisational constraints shape daily work, discrepancies between formal policies and actual practice may be particularly pronounced [1,2,3]. By capturing this experiential dimension, the DWS-N extends existing instruments, such as the Decent Work Scale and the Decent Work Questionnaire, which primarily assess general working conditions rather than profession-specific processes through which such conditions are realised in practice [28,29].

### 4.3. Relationship with the JD-R Framework and Proactive Behaviours

The observed associations between Decent Work and work engagement, burnout, and job crafting provide further support for the theoretical relevance of the scale. Higher levels of Decent Work were associated with increased work engagement and reduced burnout, consistent with the Job Demands-Resources (JD-R) model [10,11].

Importantly, the results of the multiple regression analyses suggest that Decent Work does not operate as a uniform construct but rather influences outcomes through differentiated pathways. Specifically, Meaningful and Fulfilling Work (Factor 1) showed a strong association with work engagement, whereas Appropriate Working Time and Work–Life Balance (Factor 4) showed a particularly strong association with reduced burnout.

These findings indicate that Decent Work may function as a higher-order structural resource that simultaneously contributes to both the motivational process (enhancing engagement) and the health impairment process (reducing burnout) described in the JD-R model [10,11].

The multiple regression analysis further revealed significant associations between job crafting and both Meaningful and Fulfilling Work (Factor 1, *β* = 0.20, *p* = 0.004) and Appropriate Working Time and Work–Life Balance (Factor 4, *β* = 0.17, *p* = 0.030). Although the variance explained was modest (R^2^ = 0.11), these findings suggest that proactive modification of work may be closely linked to both temporal resources, such as manageable workloads and opportunities for recovery, and the extent to which nurses perceive their work as meaningful and fulfilling. Limited temporal flexibility may constrain opportunities for proactive behaviour, whereas lower perceived meaning may be associated with reduced motivation to actively shape work experiences [31,32].

### 4.4. Implications for Nursing Practice

The findings have important implications for nursing practice and workforce management. The five factors identified in the DWS-N reflect key domains of working conditions in nursing, including meaningful work, protection of workers’ rights, working environment, working time, and compensation.

Importantly, the results suggest that improvements in a single domain, such as compensation, may not be sufficient to enhance overall well-being. Previous studies have consistently shown that long working hours and workload are strongly associated with burnout and reduced job satisfaction [5,6]. In line with these findings, the present results indicate that working time conditions play a critical role in reducing burnout.

From a practical perspective, the DWS-N may serve as a useful tool for assessing and monitoring working conditions in healthcare settings, identifying priority areas for intervention, and informing organisational policies. By focusing on how structural conditions are experienced by nurses, the scale provides actionable insights that go beyond the evaluation of formal policies alone. In particular, the DWS-N may help hospital managers and policymakers identify domains requiring improvement and evaluate the effectiveness of initiatives aimed at promoting nurses’ well-being and workforce retention.

### 4.5. Limitations and Future Research

This study has several limitations. First, the sample was limited to nurses working in large-scale acute care hospitals in Japan, which may restrict generalisability to other settings. In addition, response rates differed between Phases 2 and 3. This variation may reflect differences in participating institutions and sampling conditions, as Phase 2 involved large acute care hospitals in metropolitan areas, whereas Phase 3 employed an independent nationwide sample that included hospitals with varying characteristics. However, many of the structural challenges identified, such as long working hours and staffing shortages, are widely observed across healthcare systems [1,2,3], suggesting broader relevance.

Second, all variables were assessed using self-report measures, which may introduce common method bias. Although statistical checks indicated that this was unlikely to substantially affect the results, future studies should incorporate multiple data sources.

Finally, owing to the cross-sectional design of this study, explicit causal relationships cannot be inferred. In addition, although the cross-validation analysis demonstrated acceptable overall model fit, the GFI and AGFI values were slightly below conventional thresholds, suggesting that further validation in diverse healthcare settings and populations is warranted. Future research using longitudinal or diary study designs is needed to examine the long-term dynamics between Decent Work and workforce outcomes.

## 5. Conclusions

This study developed and validated the Decent Work Scale for Hospital Nurses (DWS-N) using a multi-phase methodological design. The findings demonstrated that the scale has a stable five-factor structure, satisfactory reliability, and acceptable validity across independent samples.

A key contribution of this study is the conceptualisation of Decent Work as the experiential realisation of structural labour conditions. By capturing not only the presence of organisational provisions but also how they are experienced by nurses in daily practice, the DWS-N provides a context-specific and theoretically grounded measurement tool.

The results further indicate that different dimensions of Decent Work are associated with distinct aspects of occupational well-being, suggesting that targeted organisational interventions may be warranted. In particular, working time conditions and meaningful work were associated with lower levels of burnout and higher levels of work engagement and proactive job crafting.

Overall, the DWS-N offers a novel and practical approach to assessing and improving working conditions in nursing, with potential applications in workforce management, organisational policy, and healthcare quality improvement.

## Figures and Tables

**Table 1:** Factor structure of the Decent Work Scale for Hospital Nurses (*n* = 356).

Cronbach’s α (Total Scale) = 0.896	Factor Loadings (F1–F5)				
	Factor 1	Factor 2	Factor 3	Factor 4	Factor 5
Factor 1: Meaningful and Fulfilling Work (α = 0.875)					
113 My job enables me to utilize my strengths	0.860	−0.140	−0.088	0.041	−0.042
109 My job is interesting and challenging	0.792	−0.022	0.000	0.047	−0.025
131 Through my work, I can achieve personal growth	0.783	−0.005	0.045	−0.055	0.016
106 My job matches my abilities	0.767	−0.008	−0.048	0.099	−0.064
103 I feel fulfilled in my work	0.659	0.042	−0.048	0.241	−0.061
Factor 2: Protection of Workers’ Rights (α = 0.830)					
93 My workplace respects individual opinions and positions	−0.044	0.872	0.034	−0.077	−0.019
80 I feel free to express my opinions at work	−0.068	0.775	−0.022	0.094	0.038
89 I can consult supervisors or senior staff when I encounter difficulties	0.059	0.658	0.008	0.018	0.011
96 I feel psychologically safe interacting with colleagues	0.056	0.628	−0.030	0.005	−0.008
127 Staff can participate fairly in decision-making related to work	−0.048	0.571	0.198	−0.011	−0.017
Factor 3: Supportive and Safe Working Environment (α = 0.725)					
47 My hospital addresses mental health issues	−0.034	0.017	0.624	0.141	0.063
51 Occupational safety goals are clearly communicated	−0.032	0.046	0.570	−0.022	0.035
75 I have a clear understanding of my working conditions	0.080	−0.155	0.558	0.036	−0.062
40 My hospital promotes work–life balance	−0.038	0.024	0.525	0.141	0.073
57 Training on violence and harassment is provided	−0.049	0.001	0.521	0.018	−0.056
23 My workplace takes measures to reduce long working hours	0.000	0.078	0.460	0.064	−0.031
Factor 4: Appropriate Working Time and Work–Life Balance (α = 0.830)					
45 I have sufficient time for hobbies and personal development	−0.048	0.007	0.080	0.806	0.035
42 I have enough time to spend with my family	−0.035	−0.038	0.078	0.712	0.044
46 I feel that my current job allows me to maintain my quality of life	0.024	0.000	0.084	0.688	0.085
Factor 5: Adequate Compensation (α = 0.838)					
1 I receive adequate pay for my work	−0.092	−0.017	−0.006	−0.011	0.874
2 My salary enables me to live the life I desire	0.086	−0.128	−0.039	0.068	0.825
3 I can plan my future without financial concerns	0.040	−0.068	−0.046	0.078	0.715

Note. Factor loadings are presented for each item. The conceptual framework of this scale was informed by the Decent Work Agenda proposed by the International Labour Organization.

**Table 2:** Participant characteristics (*n* = 247).

Variable	Category	*n* (%)
Sex	Male	38 (15.4)
	Female	208 (84.6)
Age group (years)	20–29	57 (23.2)
	30–39	74 (30.1)
	40–49	69 (28.0)
	50–59	32 (13.0)
	≥60	14 (5.7)
Years of nursing experience	2–5	39 (16.7)
	6–10	47 (20.2)
	11–15	44 (18.9)
	16–20	34 (14.6)
	21–25	28 (12.0)
	26–30	17 (7.3)
	≥31	24 (10.3)
Years of tenure	1–5	57 (26.4)
	6–10	51 (23.6)
	11–15	34 (15.7)
	16–20	30 (14.0)
	21–25	15 (6.9)
	26–30	18 (8.3)
	≥31	11 (5.1)
Partner status	Yes	154 (62.6)
	No	92 (37.4)
Children	Yes	128 (52.0)
	No	118 (48.0)
Caregiving experience	Yes	54 (22.0)
	No	192 (78.0)
Certification	Yes	10 (4.1)
	No	235 (95.9)
Work schedule	Day shift only (temporary)	7 (2.9)
	Two-shift system	109 (44.6)
	Three-shift system	117 (48.0)
	On-call duty	1 (0.4)
	Irregular schedule	10 (4.1)
Workplace childcare availability	Yes	186 (76.2)
	No	58 (23.8)
Paid leave availability	Available	186 (76.2)
	Not available	58 (23.8)

Note: Values are presented as *n* (%).

**Table 3:** Concurrent validity of the Decent Work Scale for Hospital Nurses (*n* = 247; CBI: *n* = 232).

Measure	F1 (5 Items) Meaningful and Fulfilling Work	F2 (5 Items) Protection of Workers’ Rights	F3 (6 Items) Supportive and Safe Working Environment	F4 (3 Items) Appropriate Working Time and Work–Life Balance	F5 (3 Items) Adequate Compensation	Total (22 Items)
UWES (17 items)	0.51 **	0.32 **	0.36 **	0.26 **	0.22 **	0.46 ***
CBI (19 items)	−0.45 **	−0.34 **	−0.31 **	−0.52 **	−0.38 **	−0.53 ***
JC (21 items)	0.27 **	0.22 **	0.19 *	0.25 **	0.09	0.28 ***

Note. UWES = Utrecht Work Engagement Scale; CBI = Copenhagen Burnout Inventory; JC = Job Crafting. *** *p* < 0.001, ** *p* < 0.01, * *p* < 0.05 (two-tailed).

**Table 4:** Multiple regression analysis examining associations between DWS-N subscales and occupational outcomes (*n* = 247).

	Work Engagement (UWES): R = 0.55, R^2^ = 0.30				Burnout (CBI): R = 0.60, R^2^ = 0.36				Job Crafting (JC): R = 0.33, R^2^ = 0.11			
DWS-N Subscales	*β*	*B*	*SE*	95% CI	*β*	*B*	*SE*	95% CI	*β*	*B*	*SE*	95% CI
F1 Meaningful and Fulfilling Work	0.43 ***	2.41	0.34	[1.74, 3.07]	−0.27 ***	−36.48	8.22	[−52.70, −20.27]	0.20 **	0.76	0.26	[0.25, 1.27]
F2 Protection of workers’ rights	0.02	0.08	0.30	[−0.51, 0.67]	0.08	9.80	7.24	[−10.46, 18.06]	0.05	0.13	0.23	[−0.32, 0.58]
F3 Supportive and Safe Working Environment	0.21 **	0.85	0.28	[0.31, 1.39]	−0.07	−7.25	6.70	[−20.46, 5.97]	0.04	0.12	0.21	[−0.29, 0.54]
F4 Appropriate Working Time and Work–Life Balance	−0.01	−0.03	0.41	[−0.84, 0.78]	−0.36 ***	−51.59	9.93	[−71.15, −32.02]	0.17 *	0.69	0.32	[0.07, 1.31]
F5 Adequate compensation	0.02	0.10	0.37	[−0.63, 0.84]	−0.10	−15.29	9.15	[−33.32, 2.74]	−0.09	−0.36	0.29	[−0.93, 0.21]

Note. *β* = standardized coefficient; *B* = unstandardized coefficient; *SE* = standard error; CI = confidence interval. *** *p* < 0.001, ** *p* < 0.01, * *p* < 0.05.

## Data Availability

The original contributions presented in this study are included in the article. The Decent Work Scale for Hospital Nurses (DWS-N) is available from the corresponding author upon reasonable request. Researchers who wish to use, reproduce, translate, or adapt the DWS-N must obtain prior permission from the corresponding author to ensure its appropriate application and preserve the conceptual and psychometric integrity of the instrument.

## Public Involvement Statement

No public involvement in any aspect of this research.

## Guidelines and Standards Statement

This study was conducted in accordance with COSMIN (COnsensus-based Standards for the selection of health Measurement INstruments) guidelines for scale development and validation research.
